# DVH analysis using a transmission detector and model‐based dose verification system as a comprehensive pretreatment QA tool for VMAT plans: Clinical experience and results

**DOI:** 10.1002/acm2.12743

**Published:** 2019-10-11

**Authors:** Ahamed B. Mohamed Yoosuf, Salem AlShehri, Abdulrahman Alhadab, Mamdooh Alqathami

**Affiliations:** ^1^ Department of Oncology Ministry of National Guard – Health Affairs Riyadh Saudi Arabia; ^2^ King Abdullah International Medical Research Center Riyadh Saudi Arabia; ^3^ King Saud bin Abdulaziz University for Health Sciences Riyadh Saudi Arabia

**Keywords:** dose volume histogram analysis, volumetric modulated arc therapy, model‐based verification system, ionization‐based transmission detector

## Abstract

**Purpose:**

Dose volume histogram (DVH)‐based analysis is utilized as a pretreatment quality assurance tool to determine clinical relevance from measured dose which is difficult in conventional gamma‐based analysis. In this study, we report our clinical experience with an ionization‐based transmission detector and model‐based verification system, using DVH analysis, as a comprehensive pretreatment QA tool for complex volumetric modulated arc therapy plans.

**Methods and Materials:**

Seventy‐three subsequent treatment plans categorized into four clinical sites (Head and Neck, Thorax, Abdomen, and Pelvis) were evaluated. The average dose (D_mean_) and dose received by 1% (D_1_) of the planning target volumes (PTVs) and organs at risks (OARs) calculated using the treatment planning system (TPS) were compared to a computed (model‐based) and reconstructed dose, from the measured fluence, using DVH analysis. The correlation between gamma (3% 3 mm) and DVH‐based analysis for targets was evaluated. Furthermore, confidence and action limits for detector and verification systems were established.

**Results:**

Linear regression confirmed an excellent correlation between TPS planned and computed dose using a model‐based verification system (*r* ^2^ = 1). The average percentage difference between TPS calculated and reconstructed dose for PTVs achieved using DVH analysis for each site is as follows: Head and Neck — 0.57 ± 2.8% (D_mean_) and 2.6 ± 2.7% (D_1_), Abdomen — 0.19 ± 2.8% and 1.64 ± 2.2%, Thorax — 0.24 ± 2.1% and 3.12 ± 2.8%, Pelvis 0.37 ± 2.4% and 1.16 ± 2.3%, respectively. The average percentage of passed gamma values achieved was above 95% for all cases. However, no correlation was observed between gamma passing rates and DVH difference (%) for PTVs (*r* ^2^ = 0.11). The results demonstrate a confidence limit of 5% (D_mean_ and D_1_) for PTVs using DVH analysis for both computed and reconstructed dose distribution.

**Conclusion:**

DVH analysis of treatment plan using a model‐based verification system and transmission detector provided useful information on clinical relevance for all cases and could be used as a comprehensive pretreatment patient‐specific QA tool.

## 1 INTRODUCTION

A radical development in radiotherapy treatment planning techniques combined with advanced imaging and delivery systems with various degrees of freedom allows the delivery of high doses with steep dose gradients aimed at the target while sparing surrounding normal tissues.^1^, ^2^ Volumetric modulated arc therapy (VMAT) and stereotactic body radiotherapy (SBRT) techniques are routinely used in clinical practice to treat complex targets in various treatment sites.^2^, ^3^

Due to reduced safety margin and high doses delivered in short fraction, any potential errors in planning and delivery would lead to serious consequences for patients.^1^ The dose delivery to the target is influenced by uncertainties in the planning (complexity of plans) and delivery (design of the multileaf collimators) systems.^4^ Hence, each treatment plan created using complex techniques requires a comprehensive patient‐specific pretreatment quality assurance (QA) procedure to verify the dose calculation generated in the treatment planning system (TPS) and delivery system such as linear accelerator.

Pretreatment QA methods based on films, ionization chamber or scintillation detectors, portal dosimetry, Monte Carlo and log file analysis has been published and proven useful for patient‐specific pretreatment QA, but each method has its weaknesses as well. Traditionally, independent monitor unit calculation softwares are utilized to verify the TPS dose calculation. To verify the beam delivery, 2D detector arrays equipped with ionization chambers or semiconductor detectors are commonly used and play a major role to ensure that an IMRT treatment plan is accurately delivered.^5^, ^6^, ^7^

Conventional pretreatment QA includes delivering the patient plan to a standard phantom and comparing the measured and calculated 3D dose distribution using gamma analysis with different passing criteria.^5^ The gamma index is calculated by combining the percentage dose difference and distance to an agreement for each of the pixels within the region of interest.^6^ While gamma analysis based on measurements using different detectors provides a valuable understanding of whether the linear accelerator is operating as planned, it does not provide any correlation indicating a decrease in clinical metric with increasing and decreasing passing rate nor predict the clinical impact.^4^, ^6^ Furthermore, the gamma analysis has limited accuracy in the regions of steep dose gradients. Portal dosimetry and log file analysis are also used for pretreatment dose verification. Nevertheless, it is often difficult to quantify and interpret the results in terms of dose to targets and organs at risk (OAR) using the traditional QA methods.^6^, ^7^, ^8^, ^9^

To overcome these limitations, the incorporation of dose volume histogram (DVH) information within the QA procedure, in addition to gamma passing rates, is required to provide a comprehensive patient‐specific pretreatment QA.^10^ This would offer an insight into the relevance of observed differences between measured and the TPS planned dose to the target and surrounding normal structures. Furthermore, dose verification at planning level combined with verification of delivery system is also required which in turn would result in a more comprehensive QA methodology as it consists of an independent dose calculation to verify TPS and verification of delivery systems for detrimental dose differences in target and OAR.^10^

Previous studies have reported the utilization of Compass system (IBA Dosimetry, Schwarzenbruck, Germany) along with 2D ionization chamber array (MatriXX, IBA Dosimetry) for pretreatment verification.^10^, ^11^, ^12^ The studies have reported a good agreement between the Compass computation and reconstructed dose in VMAT plans for dose calculated with Monaco (Elekta Inc., St Louis, MO, USA) and Eclipse (Varian Medical Systems Finland Oy, Helsinki, Finland) treatment planning system.^11^, ^12^ Furthermore, the accuracy of the reconstructed 3D dose distributions obtained using the Compass system and Dolphin detector has been evaluated.^13^, ^14^ However, no studies to date have reported the local confidence limits and action limits for targets and OARs in the utilization of model‐based comprehensive patient‐specific pretreatment QA for all clinical sites, with a sufficient number of cases, based on DVH analysis.

This study aimed to present the clinical experience on utilizing a model‐based QA tool and Dolphin transmission detector, using DVH‐based analysis, as a comprehensive patient‐specific pretreatment QA for complex techniques planned for different clinical sites. The correlation between calculated and delivered dose distribution for targets and OAR were studied. Furthermore, local confidence limits and action limits along with uncertainty analysis were evaluated.

## 2 METHODS

### 2.1 Treatment planning and delivery

In this prospective study, 73 subsequent VMAT clinical treatment plans treated between November 2018 and April 2019 were selected for evaluation. The cases were categorized into four clinical sites: Head and Neck (n = 19), Thorax (n = 16), Abdomen (n = 17), and Pelvis (n = 22). All treatment plans were generated using Monaco v5.11 (Elekta AB, Sweden) TPS based on Monte Carlo dose calculation algorithm using a dose grid of 2.5 mm and a nominal acceleration potential of 6 MV was used. All plans were delivered on an Infinity^®^ (Elekta, Stockholm, Sweden) linear accelerator equipped with Agility™ multileaf collimators (MLC).

### 2.2 Dose objective and constraints

The individual plan was generated to achieve better dose conformity with steep dose gradients and good target coverage while maintaining the constraints to critical organs as recommended in QUANTEC and RTOG protocols.^15^, ^16^ The OARs evaluated in this study included: Head and Neck — brainstem, mandible, oral cavity, larynx, spinal cord, eyes, optic nerves, optic chiasm, lens, cochlea, and parotid; Thorax — esophagus, lung, spinal cord, and heart; Abdomen — bowel, kidney, liver, and spinal cord; Pelvis — rectum, femoral head, bladder, and bowel. All plans were accepted and clinically approved for treatment by a Consultant Physician.

### 2.3 Compass verification system

Compass verification system v4.1 (IBA Dosimetry, Schwarzenbruck, Germany) is an integrated software solution comprising a dedicated beam model and a virtual accelerator that was created with the photon beam commissioning data of the institutional linear accelerator. Further details on the Compass system are published elsewhere.^1^, ^14^, ^17^ The dose engine implemented in Compass uses a collapsed cone convolution/superposition (CC) algorithm.^18^ The DICOM files of each treatment plan (CT image sets, RT structures, RT plans, and RT doses) were exported to the Compass verification system. The grid size used in Compass for dose computation and reconstruction is similar to the TPS plan (2.5 mm).

### 2.4 Dolphin transmission detector

Dolphin transmission detector array (IBA Dosimetry, Schwarzenbruck, Germany) consists of a pixel‐segmented ionization chamber that is a 2D array of 1513 air‐vented plane‐parallel chambers that are arranged in a square plane with an active area of 240 mm^2^ × 240 mm^2^. The Dolphin transmission detector is mounted at the gantry at a source to surface distance (SSD) of 600 mm. The size of each air‐vented chamber is 3.2 mm in diameter and 2 mm in height. The spacing of the detector is 5 mm for a field size of up to 140 mm^2^ × 140 mm^2^ which projects to approximately 8 mm in isocenter distance when the source‐to‐detector distance is 600 mm, whereas 5–10 mm in the remaining area.^13^ Previous studies have investigated the validation and error detection capability of the Compass verification system and the Dolphin detector in detail.^1^, ^14^, ^19^, ^20^

### 2.5 Evaluation metrics

A conventional global gamma analysis was performed for all cases by normalizing both calculated and measured to the maximum absolute dose from TPS. A distance to agreement (DTA) of 3 mm and dose difference of 3% with a 10% lower dose limit threshold was applied for all cases to exclude the clinically irrelevant dose levels. A passing percentage of 95% with gamma values ≤1 were applied for all cases.^21^, ^22^

For independent model‐based TPS verification, the dose calculation generated in TPS was compared to dose computed (DC) by Compass verification system using the CC algorithm. Secondly, for measurement‐based pretreatment QA, TPS calculated dose was compared to reconstructed dose (RD) generated directly on patient anatomy based on fluence measurement using Dolphin transmission detector.

The DVH‐based indices: the average dose (D_mean_) and dose received by 1% (D_1_) of the target volumes and OARs for all cases calculated using TPS was compared to Compass dose computation (DC) and reconstructed doses (RD). The results were statistically evaluated and local confidence limits were derived utilizing the concept confidence limit of |mean|+1.96σ and successively an action limit was established that account for the deviation in quality measures which requires clinical intervention.^4^ Furthermore, the correlation between gamma pass rate (3% 3 mm criteria) and mean difference, attained between planned and measured dose, using DVH analysis for PTVs was studied. The Pearson correlation coefficient was used to calculate the correlation between calculated and measured dose distribution. Furthermore, TPS achieved dose constraints for OARs in each case were compared to the reconstructed dose measured using the Dolphin detector with the Compass verification system. Uncertainty analysis was studied for a transmission detector and Compass verification system. The overall uncertainty was calculated as the square root of the sum of the squares of all the listed uncertainties.

## 3 RESULTS

The DVH parameters (D_mean_ and D_1_), averaged on 73 cases, with corresponding Pearson's correlation coefficient value for target volumes and OARs between TPS calculated and Compass computed/reconstructed doses are presented in Tables 1 and 2. The mean planned dose (D_mean_ and D_1_) using the Monte Carlo algorithm, for target volumes (PTVs) and OARs for all cases, resulted in good agreement with Compass computed dose calculated using CC algorithm (*r* ^2^ = 1). This ensured an independent dose verification of TPS calculation.

**Table 1:** Comparison of TPS calculated dose (mean) for target volumes and OARs to reconstructed (measured using Dolphin detector) and Compass computed dose (CC algorithm).

Sites	TPS — Reference (cGy) D_mean_ (range)	Reconstructed dose (cGy) D_mean_ (range)	Correlation coefficient (*r* ^2^)	Computed dose (cGy) D_mean_ (range)	Correlation coefficient (*r* ^2^)
Thorax: Pres. dose (2000–6000 cGy)					
PTV	4981 (3120–6170)	4959 (3225–6270)	0.99	4759 (3114–6151)	1.00
Esophagus	2085 (199–3574)	2088 (200–3682)	1.00	2083 (193–3553)	1.00
Lung	954 (131–2494)	960 (123–2623)	0.99	949 (130–2523)	1.00
Spinal cord	1214 (482–2724)	1304 (559–3077)	0.99	1232 (511–2735)	1.00
Heart	769 (155–1622)	734 (137–1500)	0.95	779 (175–1609)	1.00
Head & neck: Pres. dose (1980–7000 cGy)					
PTV	5110 (813–7552)	5082 (829–7297)	0.98	5105 (807–7.46)	1.00
Brainstem	2263 (174–5149)	2359 (314–6053)	0.96	2290 (210–5130)	1.00
Mandible	3891 (648–5802)	4360 (717–6402)	1.00	4048 (654–5919)	1.00
Oral cavity	3028 (132–4859)	3329 (129–5513)	1.00	3094 (156–4902)	1.00
Larynx	3456 (575–4595)	3876 (612–5935)	0.97	3498 (577–4650)	1.00
Eyes	815 (101–2867)	1046 (113–3451)	0.95	878 (131–3052)	0.99
Optic nerve	1864 (114–4779)	2031 (138–5284)	0.92	1890 (138–4842)	1.00
Lens	634 (106–1954)	951 (148–3472)	0.94	742 (131–2300)	0.99
Optic chiasm	2034 (105−5136)	2263 (126–5383)	0.89	2060 (130–5066)	1.00
Cochlea	2207 (137–5086)	2599 (168–6399)	0.97	2366 (162–5397)	1.00
Parotid	2823 (642–6109)	3347 (471–6784)	0.98	2925 (675–6248)	0.99
Abdomen: Pres. dose (1000–6000 cGy)					
PTV	3935 (513–6238)	3952 (533–6752)	1.00	3708 (157–6376)	1.00
Spinal cord	1427 (105–2752)	1475 (102–2833)	1.00	1401 (104–2718)	1.00
Bowel	2157 (402–3708)	2206 (422–3757)	1.00	2140 (410–3633)	1.00
Kidney	1074 (137–1859)	1101 (149–1844)	0.99	1080 (136–1848)	1.00
Liver	1349 (137–2152)	1483 (154–2304)	1.00	1314 (138–2136)	1.00
Pelvis: Pres. dose (1800–6000 cGy)					
PTV	4812 (1017–6307)	4841 (939–6277)	1.00	4741 (992–6206)	1.00
Rectum	3538 (269–4869)	3583 (247–4990)	1.00	3490 (275–4788)	1.00
Femoral head	1945 (191−3957)	1921 (177–3853)	0.97	1943 (216–3933)	1.00
Bladder	3804 (521–5599)	3951 (350–5854)	0.99	3746 (512–5470)	1.00
Bowel	1698 (169–3885)	1918 (199–4455)	0.97	1689 (168–3864)	1.00

Abbreviations: OAR, organs at risks; PTV, planning target volume; TPS, treatment planning system.

**Table 2:** Comparison of TPS calculated dose (D1) for target volumes and OARs to reconstructed (measured using Dolphin detector) and Compass computed dose (CC algorithm).

Sites	TPS — Reference (cGy) D_1_ (range)	Reconstructed dose (cGy) D_1_ (range)	Correlation coefficient (*r* ^2^)	Computed dose (cGy) D_1_ (range)	Correlation coefficient (*r* ^2^)
Thorax: Pres. dose (2000–6000 cGy)					
PTV	5269 (3268–6557)	5498 (3530–7202)	0.99	5247 (3293–6485)	1.00
Esophagus	4218 (633–5695)	4279 (613–5736)	1.00	4176 (617–5653)	1.00
Lung	3663 (1157–6423)	3717 (1240–6906)	1.00	3630 (1154–6402)	1.00
Spinal cord	2470 (1078–5146)	2661 (1260–5428)	0.99	2471 (1091–5207)	1.00
Heart	3442 (658–4923)	3150 (497–4464)	0.97	3442 (658–4924)	1.00
Head & neck: Pres. dose (1980–7000 cGy)					
PTV	5507 (926–7692)	5709 (949–8065)	1.00	5510 (919–7826)	1.00
Brainstem	3774 (644–5908)	3912 (695–6803)	0.91	3835 (645–5865)	0.99
Mandible	5415 (811–6874)	5807 (851–7606)	1.00	5485 (803–6990)	1.00
Oral cavity	2755 (132–4859)	3031 (129–5513)	1.00	2816 (156–4902)	1.00
Larynx	3456 (575–4595)	3876 (612–5935)	0.97	3498 (156–4902)	1.00
Eyes	1600 (151–4804)	1970 (247–5808)	0.91	1621 (193–4744)	1.00
Optic nerve	2529 (136–5577)	2677 (165–6252)	0.91	2540 (165–5545)	1.00
Lens	797 (136–2556)	1289 (209–5402)	0.95	892 (149–2994)	0.99
Optic chiasm	2548 (128–5414)	2677 (154–6158)	0.92	2551 (154–5367)	1.00
Cochlea	2555 (148–5458)	2876 (184–6864)	0.97	2690 (176–5719)	1.00
Parotid	4499 (819–7418)	4715 (823–7732)	0.99	4461 (806–7387)	1.00
Abdomen: Pres. dose (1000–6000 cGy)					
PTV	4159 (536–6928)	4235 (558–7490)	1.00	3916 (472–6987)	1.00
Spinal cord	2340 (219–4077)	2381 (232–4147)	1.00	2325 (221–4067)	1.00
Bowel	4128 (1979–5160)	4266 (2151–5207)	0.99	4056 (1929–5070)	1.00
Kidney	2615 (444–4587)	2648 (513–4338)	0.98	2582 (444–4491)	1.00
Liver	3594 (518–4774)	3623 (541–4813)	1.00	3526 (517–4687)	1.00
Pelvis: Pres. dose (1800–6000 cGy)					
PTV	4952 (1089–6565)	5011 (1046–6542)	1.00	4882 (1051–6390)	1.00
Rectum	4718 (658–6065)	4864 (659–6688)	0.98	4620 (663–5962)	1.00
Femoral head	3731 (1005–4999)	3778 (863–5798)	0.94	3707 (1083–4946)	1.00
Bladder	5046 (1039–6523)	5176 (1012–6889)	0.97	4932 (1004–6350)	1.00
Bowel	3869 (514–5391)	4212 (736–6767)	0.97	3787 (515–5285)	1.00

Abbreviations: OAR, organs at risks; PTV, planning target volume; TPS, treatment planning system.

**Figure 1:**
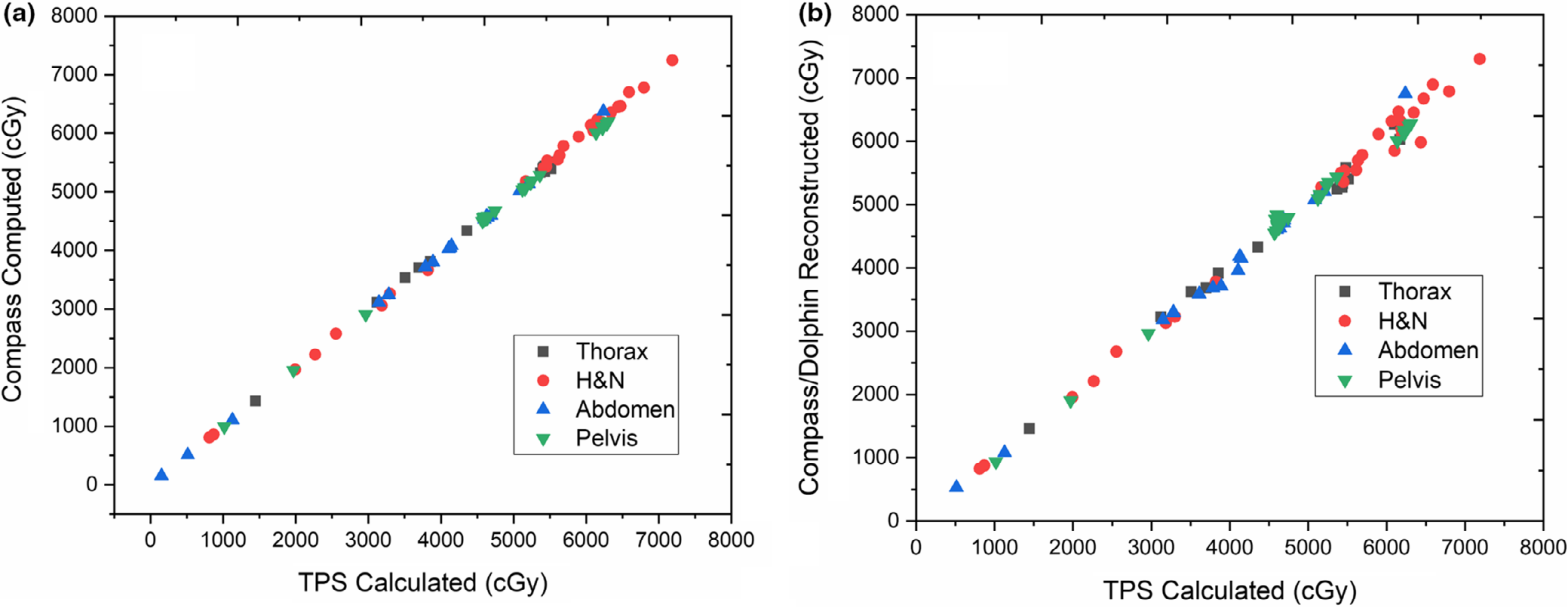
Comparison of treatment planning system calculated dose for planning target volume (mean) to: (a) Computed dose (CC algorithm) and (b) Compass reconstructed (measured using Dolphin detector).

As shown in Table 1, a good correlation was observed between TPS planned dose (mean) and Compass reconstructed dose (using Dolphin detector) for target volumes. Figure 1 illustrates the comparison of measured and calculated dose to PTVs for all cases. A marginally reduced PTV coverage was observed for Head and Neck cases from the reconstructed doses to those planned in TPS (*r* ^2^ = 0.98). Likewise, the reconstructed dose for individual OARs correlated well with TPS planned dose for all sites except small structure in Head and Neck cases and heart in thorax. The dose measured using Dolphin detector in Head and Neck cases was found to be slightly higher than TPS planned doses but mostly within the institution accepted tolerances. It was observed more so for OARs that were small in size and adjacent to or within PTVs. A similar trend was observed for D_1_ of targets and OARs between planned and measured dose using the Dolphin detector as presented in Table 2.

**Figure 2:**
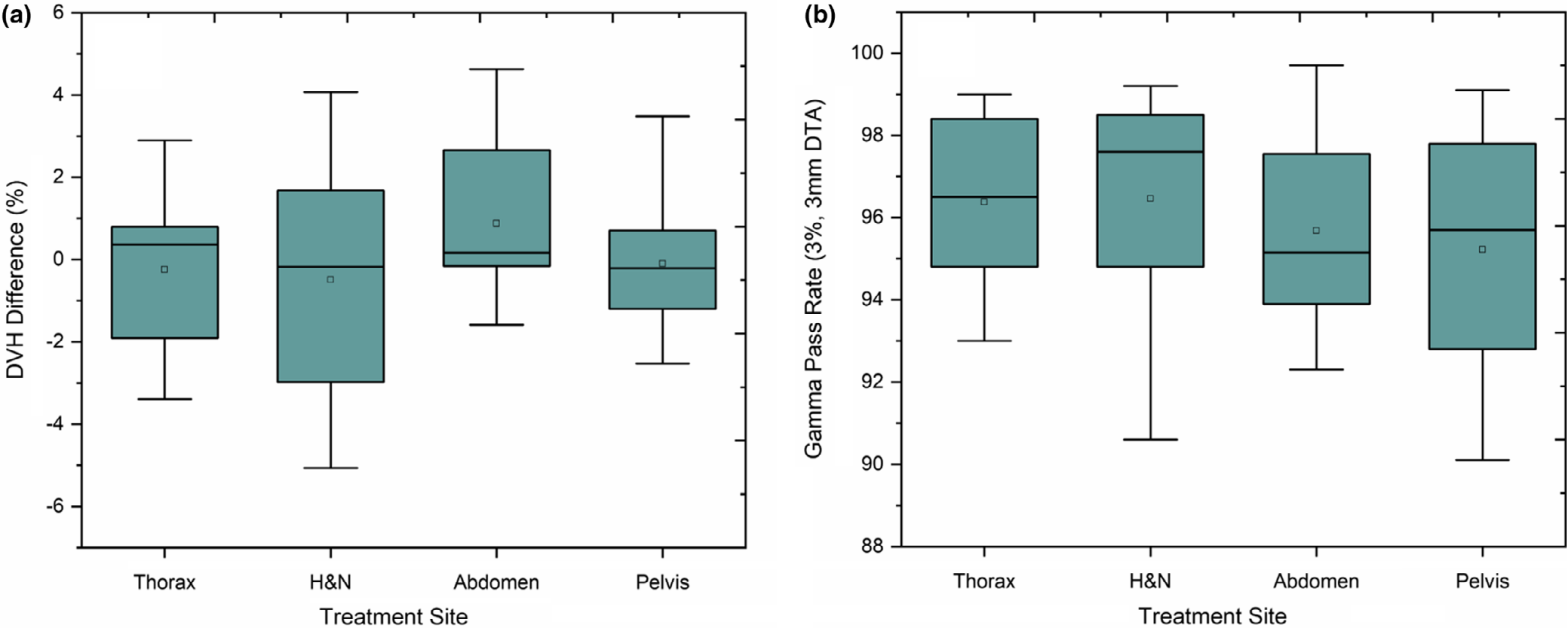
Patient number versus (a) Mean dose difference for planning target volume using dose volume histogram analysis and (b) Gamma passing rates (3%/3 mm).

The average percentage difference between TPS calculated and reconstructed dose for PTVs (D_mean_ and D_1_) achieved using DVH analysis for each site is as follows: Head and Neck — 0.57 ± 2.8% (D_mean_) and 2.6 ± 2.7% (D_1_), Abdomen — 0.19 ± 2.8% and 1.64 ± 2.2%, Thorax — 0.24 ± 2.1% and 3.12 ± 2.8%, Pelvis 0.37 ± 2.4% and 1.16 ± 2.3%, respectively. Figure 2 illustrates the gamma passing rates using 3%/3 mm criteria and the DVH percentage difference for PTVs between TPS calculated and reconstructed dose distribution. The average percentage of passed gamma values achieved was above 95% for all cases (Head and Neck — 96.5 ± 2.6%, Abdomen — 95.68 ± 2.6%, Thorax — 96.4 ± 2.4%, Pelvis 95.2 ± 2.4%). However, no correlation was observed between gamma passing rates and DVH difference (%) for the target volumes (*r* ^2^ = 0.11).

The overall confidence limit for all sites was determined to be 5% (D_mean_ and D_1_) for evaluating targets (PTV) using DVH analysis. The maximum difference observed for each case between the TPS calculated dose to the PTVs (D_mean_ and D_1_) and the computed/reconstructed dose, using Compass and Dolphin detector, was within the confidence limit of 5%. Likewise, a confidence limit of 5% (D_mean_ and D_1_) were determined for OARs of all sites (Table 1) except for a few in Head and Neck cases, which are small in volumes or received very low dose (e.g., eye, lens, cochlea, parotid, optic nerves). A small deviation in dose to these structures resulted in large percentage differences. The action limit for targets and majority of the OARs were determined to be 7%.

### 3.1 Evaluation of uncertainty

In this work, as the comparison of TPS planned and calculated dose distribution using Dolphin detector was considered the end result, standard uncertainty (a combination of type A and type B) was estimated as shown in Table 3. The square root of the sum of squares of all uncertainties was used to calculate the overall uncertainty and is estimated as 4.3% for dose computation and 5.0% for the measured (reconstructed) dose distribution.

**Table 3:** Estimated standard uncertainty for Dolphin detector and Compass verification system.

Source of uncertainty	Standard uncertainty (k = 1)		References
	Dose computation (%)	Dose reconstruction (%)	
Absolute dose and geometric calibration	0.28	0.28	25
Linac output	NA	0.5	25
Dose calculation algorithm	2.4	3.2	26
Density difference	2	2	26
Dose linearity	NA	0.7	20
Dose rate dependence	NA	0.3	20
MLC test cases	2	2	17
Dosimetric fluctuations due to detector inhomogeneity	NA	1	20
Repeatability	1	1	26
DVH‐based indices	2	2	1
Total uncertainty	4.3	5.0	

Abbreviations: DVH, dose volume histogram; MLC, multileaf collimators.

## 4 DISCUSSION

Pretreatment patient‐specific QA is widely used as the core component of most QA programs that involves complex treatment planning and delivery to combat the errors related to planning and delivery system like linear accelerator.^4^ The patient‐specific QA based on 2D array detectors is a clinically proven method for VMAT and stereotactic dose delivery. The conventional patient‐specific QA methods using gamma pass rates might provide acceptable passing rates but limited in terms of clinical impact and outcomes.^7^

This work aimed to report our clinical experience of a transmission‐based detector (Dolphin) and Compass verification system as a comprehensive patient‐specific pretreatment QA tool. The advantage of utilizing the Compass verification system is that it can act as an independent secondary TPS verification tool utilizing the CC algorithm for dose computation. Furthermore, patient‐specific measurements are performed inside the patient's anatomy as opposed to other QA tools in which the doses are calculated in the phantom.^23^ The absolute dose and geometric calibration for Dolphin detector are not needed for every measurement due to low dependency of ionization chambers within the detector and it also provides a robust QA which includes the daily variation of absolute dose of the linear accelerator.^17^, ^24^, ^25^

In general, the DVH‐based dose evaluation provides a quantitative analysis between TPS planned and computed/reconstructed dose distribution for targets and OARs for all sites with a possibility of a better interpretation of clinical impact. The DVH‐based analysis using Compass, constructed on measurement in the patient geometry, allows a clinical decision based on quantitative analysis for each structure. As reported in previous studies, the study found no correlation between the DVH‐based analysis and gamma pass rates.^4^, ^6^, ^7^, ^8^, ^9^ The evaluation of D_1_ is emphasized as the constraints for the majority of the OARs are fixed at a dose to smaller volumes. The confidence limit used in this study was determined based on the mean difference between the measured and expected values. The discrepancies between the average dose to PTVs and majority of the OARs (D_mean_ and D_1_) calculated by TPS and the Compass dose computation/reconstruction were well within the confidence limit of 5%. Large disagreement for OARs in Head and Neck cases was observed for small structures that were close to or within targets but were within the tolerance thresholds. This is attributed to detector resolution, limitation in dose calculation by CC algorithm in high‐density regions, and the electron contamination resulting from collimators and flattening filters as the detector is placed at 60 cm SSD.^2^, ^23^, ^26^ We suggest an institutional‐based protocol in evaluating these structures. Locally derived confidence limits provide a baseline values in determining the action limits which should indicate the process performance. Action limits could vary based on institution protocol, equipment, techniques, personnel experience. Furthermore, clinical intervention is strongly suggested when the dose difference between calculated and measurement exceeds action limits for PTVs and OARs, as per institution protocols. If the difference falls between the confidence and action limits, it is up to the institution protocol to decide clinical intervention. Finally, it is important to emphasize that DVH analysis based on measurement should be included as part of physics second check for all cases and any significant deviation between planned and calculated dose distribution requires further investigation.

### 4.1 Limitations

The accuracy of ionization detector, when compared to QA systems like film dosimetry, is subject to uncertainties due to volume averaging, geometrical resolution, and self‐attenuation which lead to concern about their sensitivity. Furthermore, as the Dolphin detector is attached on the head of linear accelerator, errors related to gantry, collimator, and table rotation cannot be detected. To mitigate the detector resolution limitation, a Monte Carlo generated response function for each ion chamber is applied in Compass.^23^ Furthermore, online measurement using the Dolphin detector is not within the scope of this study. Moreover, the study is limited to treatment planning and delivery system from a single institution. This warrants a multi‐institutional analysis utilizing different treatment planning and delivery systems.

DVH‐based analysis, for targets and OARs, using the Dolphin detector and Compass verification system has been demonstrated as a comprehensive tool for patient‐specific pretreatment QA. It has been showed that DVH‐based analysis provides a better interpretation of the dose distribution within the targets and OARs in case the involvement of a physician is needed for any action before the patient treatment. Furthermore, local confidence limits and action limits based on DVH differences in the PTVs and OARs, for dose computed and reconstructed using Dolphin detector, on the patient anatomy established for routine patient‐specific pretreatment QA.

## 5 CONCLUSIONS

DVH analysis of complex treatment plan using a model‐based verification system (Compass) and Dolphin transmission detector provided useful information on clinical relevance for all cases and could be used as a comprehensive pretreatment patient‐specific QA tool. Local confidence and action limits based on the average dose difference in PTVs and OARs were established for clinical QA.

## CONFLICT OF INTEREST

The authors declare that there is no conflict of interest regarding the publication of this article.
